# Dual targeting of HER2-positive breast cancer with trastuzumab emtansine and pertuzumab: understanding clinical trial results

**DOI:** 10.18632/oncotarget.25739

**Published:** 2018-08-07

**Authors:** Alberto Ocaña, Eitan Amir, Atanasio Pandiella

**Affiliations:** ^1^ Translational Research Unit, Albacete University Hospital, and CIBERONC, Albacete, Spain; ^2^ Division of Medical Oncology and Hematology, Princess Margaret Cancer Centre, University of Toronto, Toronto, Canada; ^3^ Cancer Research Center, CSIC-IBSAL and CIBERONC, Salamanca, Spain

**Keywords:** breast cancer, HER2, trastuzumab, pertuzumab, TDM1

## Abstract

Targeting of HER2-positive tumors with trastuzumab has shown to improve survival in early stage and advanced breast cancer. The addition of pertuzumab, another anti-HER2 antibody, to trastuzumab-containing regimens has demonstrated a modest increase in disease-free survival in the adjuvant setting. Unexpectedly, when pertuzumab was explored in combination with the antibody-drug conjugate TDM1 in the metastatic setting, no additional benefit was observed compared with dual targeting of HER2 with pertuzumab and trastuzumab, together with chemotherapy. Similar results were observed when exploring pathologic complete response in the neoadjuvant setting. In this article, we discuss basic science and translational data that may explain the limited efficacy observed with the combination of TDM1 and pertuzumab, including tumor heterogeneity, clonal selection, bystander effect or downregulation of the receptor by competitive binding. In addition, we review ongoing studies that could help to understand these findings.

## INTRODUCTION

Treatment advances that dramatically change outcomes in malignant disease continue to be uncommon. Many promising anti-cancer drugs may be “lost in translation” between pre-clinical and clinical research, although there are exceptions. Drugs that target the HER2 pathway in breast cancer (BC) provide examples in which early signals found on the laboratory bench reached patients’ bedside.

The discovery of the potent oncogenic properties of the HER2 transmembrane tyrosine kinase, followed by its identification as a poor prognostic marker in women whose tumors overexpress HER2, opened the possibility for its therapeutic targeting. This led to the subsequent development of trastuzumab, an antibody that targets subdomain IV in the extracellular region of HER2 [1]. Patients with *ERBB2* oncogene amplification or HER2 receptor overexpression (HER2-positive) experience a substantial improvement in survival with trastuzumab-based therapy. Women with metastatic HER2-positive BC receiving anti-HER2 therapy now have a median survival of more than 50 months with an estimated 5 year survival of 34%, a substantial improvement from results observed 20 years ago [2]. The addition of pertuzumab, an antibody that targets the dimerization arm located in subdomain II of the extracellular region of HER2, to trastuzumab and a taxane as first line treatment, provides an improvement in median survival of more than a year in women with HER2-positive metastatic breast cancer [3]. In women with localized HER2-positive disease, the addition of trastuzumab to adjuvant chemotherapy significantly and substantially improves disease-free survival (DFS) and overall survival [4]. However, the addition of pertuzumab to a trastuzumab-containing adjuvant regimen improves DFS only modestly, a finding seen only in women with the poorest outcomes (e.g. lymph node positive disease) [5].

Drugs that target HER2 are usually tolerated well (especially trastuzumab and pertuzumab), but toxicity arises with and without concurrent use of chemotherapy [6]. Antibody-drug conjugates have been developed, such as trastuzumab emtansine (also known as T-DM1), with the intention of delivering anti-HER2 treatment with a cytotoxic conjugate that has reduced side-effects compared to traditional systemic cytotoxic agents. T-DM1 is an antibody-drug conjugate (ADC) in which trastuzumab is coupled to a derivative of the potent inhibitor of microtubule polymerization maitansine: it enables the delivery of the cytotoxic drug to cells expressing HER2 with potential to increase therapeutic index and decrease off-target effects. In the registration clinical trial comparing T-DM1 to capecitabine and lapatinib, T-DM1 significantly improved progression-free (PFS) and overall survival in women who had previously received trastuzumab and a taxane for the treatment of metastatic disease [7]. T-DM1 was also found to be superior to any physician’s choice in heavily pre-treated patients with prior exposure to trastuzumab and lapatinib [8]. Women who received the ADC experienced a more favorable safety profile in comparison to the control arm, raising the possibility that T-DM1 might displace trastuzumab-based chemotherapy regimens. However, a much more modest benefit of T-DM1was seen in pertuzumab pre-treated patients [9].

Since pertuzumab increased the efficacy of trastuzumab-based regimens for women with metastatic BC, it was hypothesized that the combination of pertuzumab and T-DM1 could provide superior clinical benefits than T-DM1 alone. T-DM1 and the combination of T-DM1 plus pertuzumab (T-DM1-P) were compared to trastuzumab and a taxane (HT) as first-line treatment in patients with HER2 positive metastatic BC in the MARIANNE phase III study. In this study, neither of the T-DM1 arms showed significant improvement in PFS, the primary endpoint, compared to control HT arm, although T-DM1 arms demonstrated a safer toxicity profile [10]. Dual HER2 targeting was also investigated in the KRISTINE study, in which women with localized operable BC were randomized to receive neoadjuvant docetaxel, carboplatin, trastuzumab and pertuzumab (TCH-P) or experimental T-DM1-P. Upon evaluation of pathologic complete response, T-DM1-P was inferior to TCH-P [11]. Thus the combination of T-DM1 and pertuzumab does not appear superior to pertuzumab and trastuzumab given with chemotherapy in the treatment of advanced or early-stage BC. Could these results have been predicted from preclinical experience?.

### Tumor heterogeneity in HER2 expression

Breast cancer is a heterogeneous disease which likely reflects clonal evolution of tumor cells [12]. In addition, BC is characterized often by genetic and epigenetic changes that mediates a gain of function of a specific subpopulation [12]. Therefore even small populations of cells can have a great impact on cancer progression and be responsible for tumor relapse or resistance to treatment. Such clones could be pre-existing subpopulations or develop over time due to selective pressure from treatment [13, 14]. HER2 positive breast cancer is defined by the College of American Pathologists as complete circumferential membrane staining in more than 10% of the tumor cells [15]. Therefore, some cells and subpopulations of tumor may not over-express the protein.

The treatment strategies used in MARIANNE and KRISTINE are based on dual antibody-mediated targeting of HER2 plus a chemotherapeutic agent or conjugate targeting tubulin. Taxanes (paclitaxel and docetaxel) seem to act on microtubules as is the case of the maitansine conjugate. T-DM1 needs to reach the target tumor cells, bind to HER2 and penetrate into the malignant cell [16]. Maitansine alone cannot enter cells because of its positive charge, preventing the drug from crossing the cell membrane [17]. Hence T-DM1 is only effective against cells expressing HER2; there is no bystander effect from release of maitansine against cells that do not express HER2. Thus the lack of improved outcomes of T-DM1 and T-DM1-P compared with trastuzumab plus chemotherapy in the MARIANNE and KRISTINE studies could be explained partially by lack of effect of T-DM1 on cells that do not overexpress HER2 but coexist with the HER2 overexpressing cells in a heterogeneous tumour [18]. Selective pressure from treatment in a tumor with heterogeneous HER2 expression, may result in the dominance of clones with limited or no expression of HER2 [19, 20]. These cells could still be targeted by conventional chemotherapy but not by cytotoxic catabolites derived from T-DM1. The combination of T-DM1 and chemotherapy has been explored [21], and although there is acceptable efficacy in early phase trials, this approach seems highly toxic.

### Sequential use of anti-HER2 drugs

The sequence of administration of two anti-HER2 directed therapies could influence expression of the target [22, 23]. An *in-vitro* study in HER2-positive breast cancer cell lines suggests that the sequence of administration of pertuzumab and T-DM1 could influence the effect of the combination [23]. When pertuzumab was administered one hour before T-DM1, pertuzumab had an antagonistic effect on T-DM1 in some cell lines; this was not observed with concurrent treatment or with the reverse sequence (T-DM1 followed by pertuzumab). Several preclinical studies have not shown substantial down-regulation of cell surface HER2 by anti-ectodomain antibodies in HER2-overexpressing cells [24–26]. However, competitive binding and steric interference could represent potential mechanisms of resistance to this drug combination [27]. These pre-clinical results might explain the disappointing efficacy results of T-DM1-P observed in the KRISTINE and MARIANNE trials.

### Ongoing studies

There are at least 5 ongoing clinical trials exploring the combination of T-DM1-P in different BC settings (see Table 1). For each trial, it is important to consider the potential implications of tumor heterogeneity and the sequence of anti-HER2 drug administration. Special attention should be given to those where concomitant use of T-DM1 and pertuzumab is given. Some of these trials include the collection of multiple tumor samples to address inter- and intra-tumor heterogeneity. Available data do not suggest that the activity of T-DM1 or pertuzumab is restricted to certain biomarker-defined groups (see Supplementary Table 1 for prospective data and Supplementary Table 2 for retrospective data). However, the question of how HER2 and other relevant pathways (like estrogen receptor, MET, IGFR-1, c-SRC, and EphA2) may influence response is being explored, in addition to mechanisms described with resistance to T-DM1 [26, 28, 29]. Special attention regarding the mechanisms of resistance to T-DM1 should be given to the internalization of the drug within the cell, and its subsequent release from lysosomes.

**Table 1:** Currently active/ongoing clinical trials evaluating the combination of T-DM1 and pertuzumab in HER2-positive BC

Study brief title	Clinical trial phase and methodology	Eligibility	Intervention and treatment intent	Primary outcome	Secondary outcome	Study rationale	Estimated primary^*^ and final completion date
T-DM1 + Pertuzumab in Pre-OP Early-Stage HER2+ BRCA	• Phase II • Single group assignment • Open label	TNM Stage II-III HER2- positive BC	T-DM1 + pertuzumab **Intent:** neoadjuvant	pCR	• CR, DFS, OS • Safety and tolerability • Enrichment for HER2-negativity or HER2 heterogeneity in residual tumor • Intra-tumor heterogeneity of HER2 amplification	Evaluate the efficacy of T-DM1-P based on HER2-positive tumor heterogeneity	**Primary:** August 2018 **Final:** April 2022
I-SPY 2 TRIAL: Neoadjuvant and Personalized Adaptive Novel Agents to Treat Breast Cancer	• Phase III • Parallel assignment • Open label	TNM Stage II-III HER2- positive BC	T-DM1 + pertuzumab ––› weekly paclitaxel and adriamycin + cyclophosphamide **Intent:** neoadjuvant	pCR	• RFS, OS • Safety and tolerability • MRI tumor volume • Establishing predictive and prognostic indices of pCR and RCB	To determine whether adding experimental agents to standard neoadjuvant regimens increases the probability of pCR	**Primary:** December 2020 **Final:** not available
Trastuzumab and Pertuzumab Followed by T-DM1 in MBC	• Phase II • Randomized • Parallel assignment • Open Label	TNM Stage IV and recurrent HER2- positive BC	Trastuzumab, Pertuzumab and T-DM1 Vs. Trastuzumab, Pertuzumab, Paclitaxel/ Vinorelbine **Intent:** palliative	OS	• PFS 1st line. 2nd and 3rd line treatment, ORR, CBR • QoL • Safety and tolerability	To understand which is the optimal treatment strategy either in combination or in sequence with chemotherapy for metastatic HER2-positive BC	**Primary:** November 2017 **Final:** November 2019
A Study Evaluating T-DM1 Plus Pertuzumab Compared With Chemotherapy Plus Trastuzumab and Pertuzumab for Participants With HER2-Positive Breast Cancer (KRISTINE)	• Phase III • Randomized • Parallel assignment • Open label	TNM T2-cT4, cN0-cN3, cM0 HER2-positive BC	Carboplatin, docetaxel, pertuzumab and trastuzumab Vs. T-DM1 and pertuzumab. **Note:** Patients receiving TCH will receive adjuvant trastuzumab and pertuzumab while patients receiving T-DM1 and pertuzumab will receive adjuvant T-DM1 and pertuzumab. **Intent:** neoadjuvant	pCR	• EFS • iDFS • OS • % patients receiving breast conserving surgery • QoL • Metabolite/catabolite concentrations and anti-therapeutic antibodies	To evaluate the efficacy and safety of T-DM1 + pertuzumab versus chemotherapy, trastuzumab + pertuzumab in operable HER2-positive BC	**Primary:** December 2015 **Final:** March 2018
A Study of T-DM1 Plus Pertuzumab Following Anthracyclines in Comparison With Trastuzumab Plus Pertuzumab and a Taxane Following Anthracyclines as Adjuvant Therapy in Participants With Operable HER2-Positive Primary Breast Cancer	• Phase III • Randomized • Parallel assignment • Open label	TNM N1 or T2 HER2 positive BC	Anthracycline ––› trastuzumab + pertuzumab + taxane Vs. anthracycline ––› T-DM1 + pertuzumab **Intent:** neoadjuvant	iDFS	• iDFS + second non-breast cancer, DFS, DRFS, OS • QoL • Safety and tolerability	To evaluate the efficacy and safety of T-DM1 in combination with pertuzumab versus trastuzumab in combination with pertuzumab and a taxane as adjuvant regimen	**Primary:** January 2024 **Final:** Data not available

^*^Final data collection date for primary outcome measure.

Abbreviations: Breast Cancer: BC; Maximum Tolerated Dose: MTD; Dose Limiting Toxicity: DLT; Relapse Free Survival: RFS; Invasive Disease Free Survival: iDFS; Disease Free Survival: DFS; Distant Recurrence Free Survival: DRFS; Clinical Response: CR; Objective Response Rate: ORR; Clinical Benefit Rate: CBR; Overall Survival: OS; Progression Free Survival: PFS; Quality of Life: QoL; Residual Cancer Burden: RCB; Epidermal Growth Factor Receptor 2: HER2.

It is important to conduct further preclinical research on the sequence and timing of anti-HER2 agents. If efficacy depends on sequence, then new clinical trials should be designed using the optimal sequence.

In conclusion, there is substantial uncertainty about the results of studies exploring the combination of T-DM1 and pertuzumab. We hope that scientifically driven translational research in ongoing clinical studies, will provide light as to how optimize these treatments for the benefit of our patients.

## SUPPLEMENTARY MATERIALS TABLES




