# Supplementary material for: Dual targeting of HER2-positive breast cancer with trastuzumab emtansine and pertuzumab: understanding clinical trial results
Source: Oncotarget. 2018 Aug 7;9(61):31915–9. doi: 10.18632/oncotarget.25739 (PMC6112749; doi:10.18632/oncotarget.25739)
Supplement: Supplementary file 1 [file oncotarget-09-31915-s001.pdf]

# Dual targeting of HER2-positive breast cancer with trastuzumab emtansine and pertuzumab: understanding clinical trial results

## SUPPLEMENTARY MATERIALS

**Supplementary Table 1: Published studies with prospective data regarding the role of certain biomarkers on response to novel anti-HER2 agents.** See Supplementary_Table_1

**Supplementary Table 2: Published studies with retrospective data regarding the role of certain biomarkers on response to T-DM1**

| Study | Study phase | Selection criteria | Objectives | Biomarkers | Response if biomarker present |
|---|---|---|---|---|---|
| Relationship between tumor biomarkers and efficacy in TH3RESA, a phase III study of trastuzumab emtansine (T-DM1) vs. treatment of physician's choice in previously treated HER2-positive advanced breast cancer<br>Kim SB, Wildiers H, Krop IE, Smitt M, Yu R, Lysbet de Haas S, Gonzalez-Martin A.<br>Int J Cancer. 2016; 139:2336–42. https://doi.org/10.1002/ijc.30276. | III | Patients with HER2-positive advanced breast cancer who received prior taxane therapy and ≥2 HER2-directed regimens, including trastuzumab and lapatinib (advanced setting), were randomized to trastuzumab emtansine (T-DM1) or treatment of physician's choice (TPC) capecitabine plus lapatinib | Examine the relationship between HER2-related biomarkers and PFS in an exploratory analysis | HER2 and HER3 mRNA expression, PIK3CA, and PTEN | T-DM1 prolonged median PFS in all biomarker subgroups analyzed, including activating PIK3CA mutations, with numerically greater benefit in patients with tumors expressing HER2 mRNA >median vs. ≤median |
| Relationship between Tumor Biomarkers and Efficacy in EMILIA, a Phase III Study of Trastuzumab Emtansine in HER2-Positive Metastatic Breast Cancer.<br>Baselga J, Lewis Phillips GD, Verma S, Ro J, Huober J, Guardino AE, Samant MK, Olsen S, de Haas SL, Pegram MD.<br>Clin Cancer Res. 2016; 22:3755–63 | III | Patients with centrally confirmed HER2-positive, unresectable, locally advanced breast cancer or MBC who had received treatment previously with trastuzumab and a taxane. | Investigate whether the efficacy of trastuzumab emtansine (T-DM1) it's correlated with the expression of specific biomarkers in the phase III EMILIA study | HER2 ($n$ = 866), EGFR ($n$ = 832), and HER3 ($n$ = 860) mRNA expression by quantitative reverse transcriptase PCR; for PTEN protein expression ($n$ = 271) by IHC; and for PIK3CA mutations ($n$ = 259) using a mutation detection kit | T-DM1 appears to be effective in both PI3KCA-mutated and wild-type tumors |

LABC or ABC: advanced breast cancer, MBC = metastatic breast cance.
